# Risk Stratification of Sudden Cardiac Death After Acute Myocardial Infarction

**DOI:** 10.19102/icrm.2018.090201

**Published:** 2018-02-15

**Authors:** An H. Bui, Jonathan W. Waks

**Affiliations:** ^1^Beth Israel Deaconess Medical Center, Harvard Medical School, Boston, MA, USA

**Keywords:** Myocardial infarction, risk stratification, sudden cardiac death

## Abstract

Despite advances in the diagnosis and treatment of acute coronary syndromes and an overall improvement in outcomes, mortality after myocardial infarction (MI) remains high. Sudden death, which is most frequently due to ventricular tachycardia or ventricular fibrillation, is the cause of death in 25% to 50% of patients with prior MI, and therefore represents an important public health problem. Use of the implantable cardioverter-defibrillator (ICD), which is the primary method of reducing the chance of arrhythmic sudden death after MI, is costly to the medical system and is associated with procedural and long-term risks. Additionally, assessment of left ventricular ejection fraction (LVEF), which is the primary method of assessing a patient’s post-MI sudden death risk and appropriateness for ICD implantation, lacks both sensitivity and specificity for sudden death, and may not be the optimal way to select the subgroup of post-MI patients who are most likely to benefit from ICD implantation. To optimally utilize ICDs, it is therefore critical to develop and prospectively validate sudden death risk stratification methods beyond measuring LVEF. A variety of tests that assess left ventricular systolic function/morphology, potential triggers for ventricular arrhythmias, ventricular conduction/repolarization, and autonomic tone have been proposed as sudden death risk stratification tools. Multivariable models have also been developed to assess the competing risks of arrhythmic and non-arrhythmic death so that ICDs can be utilized more effectively. This manuscript will review the epidemiology of sudden death after MI, and will discuss the current state of sudden death risk stratification in this population.

## Introduction

Advances in the diagnosis and treatment of patients with acute myocardial infarction (MI) have resulted in significant reductions in total mortality over time.^1,2^ Despite these improvements, however, mortality after MI remains high, and has been reported as being between 7% and 20% at one year.^1,3^ Sudden death, most often due to ventricular tachycardia (VT) or ventricular fibrillation (VF), is the cause of death in 25% to 50% of patients with prior MI,^4–6^ and implantable cardioverter-defibrillators (ICDs) can significantly reduce the risk of arrhythmic sudden death when used in appropriate patients following MI. As ICD implantation is costly and associated with risks, a great deal of effort has been made to prospectively identify the subgroup of patients with MI who are at highest risk of arrhythmic sudden death, and whom therefore would likely derive the most benefit from ICD implantation. This manuscript will review the epidemiology of sudden death and ventricular arrhythmias post-MI, and will discuss the current state of risk stratification for and the prevention of sudden death in this population.

## Etiology and pathogenesis of sudden death post-myocardial infarction

Understanding the mechanisms of sudden death after MI is critical to understanding risk stratification tools and the utility of therapies such as the ICD in reducing the incidence of sudden death. Although ventricular arrhythmias (eg, VT and VF) are overall the most common causes of sudden death after MI, other non-arrhythmic causes of sudden death are common, especially in the early period following acute MI. The distinction between arrhythmic and non-arrhythmic sudden death is critical, as ICDs, which currently are the primary method of sudden death risk reduction, can only prevent death due to arrhythmia.

**Figure 1:**
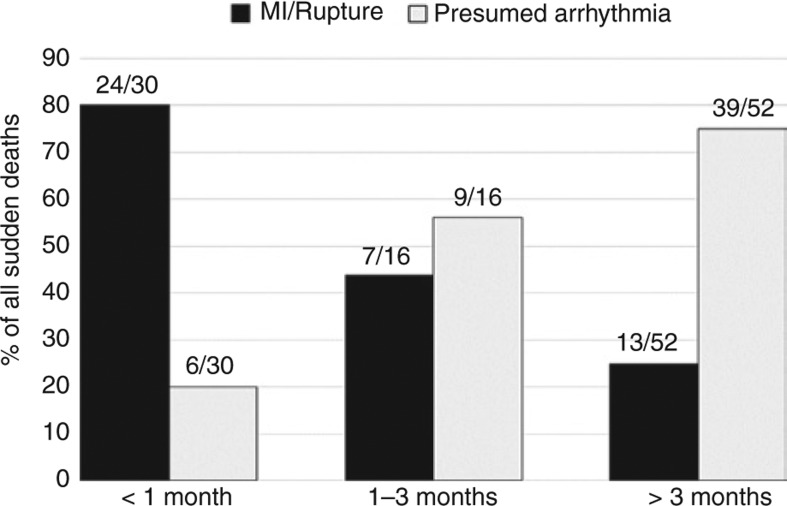
The relative number of sudden deaths that were attributed to MI/myocardial rupture versus those attributed to presumed arrhythmia occurring at various time points after MI in patients in the VALIANT trial. The rate of non-arrhythmic sudden death is highest in the early post-MI period and then decreases over time. Adapted from Pouleur AC, Barkoudah E, Uno H, et al. Pathogenesis of sudden unexpected death in a clinical trial of patients with myocardial infarction and left ventricular dysfunction, heart failure, or both. *Circulation*. 2010;122(6):597–602.

A review of autopsy records from patients in the Valsartan in Acute Myocardial Infarction Trial (VALIANT), which evaluated approximately 14,000 patients with MI, clinical heart failure, and left ventricular ejection fraction (LVEF) < 35% to 40% (depending on imaging modality), found that among 398 total autopsies and 105 sudden deaths, 27% occurred due to recurrent MI, 12% were due to cardiac rupture, 4% were due to pump failure, and 51% were due to presumed arrhythmia (essentially a diagnosis of exclusion). Additionally, the incidence of arrhythmic sudden death changed over time. In the immediate post-MI period, non-arrhythmic sudden death was much more frequent than it was many months after MI; arrhythmic sudden death accounted for 20% of sudden deaths within the first month after MI, but after three months, 75% of sudden deaths were presumed to be secondary to ventricular arrhythmias **(Figure 1)**.^7^

The pathophysiology of post-infarction VT has been well described, and involves a ventricular substrate favorable for arrhythmias and triggering events. The substrate favorable for post-MI VT involves ventricular scar and/or abnormal myocardium and resultant areas of slow conduction and conduction block created by infarction and subsequent ventricular remodeling.^8–11^ Reentrant arrhythmias usually manifest as monomorphic VT, which can further degenerate into VF. All post-MI VTs, however, are not due to reentry, and some are caused by abnormal calcium signaling and focal, triggered activity.^12^ During the acute phase of MI, ventricular arrhythmias can also occur due to alterations in cellular electrical activity, ion channel function, and abnormal transmembrane potentials within injured myocytes.^13,14^ Due to these transient cellular insults, ventricular arrhythmias occurring during this acute phase (usually within the first 24 to 48 hours after onset of MI) are associated with increased in-hospital mortality but not with increased late mortality.^15–18^ Some arrhythmias may be triggered by one mechanism and sustained by another (such as triggered activity causing premature ventricular depolarizations that then induce a reentrant VT). Heart failure itself may also trigger ventricular arrhythmias through a variety of mechanisms. Abnormalities in ventricular conduction, ventricular repolarization, and autonomic tone all influence the onset and sustainability of ventricular arrhythmias, and form some of the targets for sudden death risk stratification as will be discussed in more detail in the sections below.

## Timing and incidence of sudden death after myocardial infarction

The highest rates of total mortality and sudden death occur within the first six months after acute MI.^19,20^ Although this early period is associated with a high risk of sudden death due to arrhythmia, as previously noted, up to 50% of sudden deaths during this period may be due to non-arrhythmic causes, such as free wall rupture and recurrent MI.^7^ The VALIANT trial also demonstrated a time-dependent risk of sudden death in which the rate was found to be highest in the first 30 days (1.4% per month), after which it decreased over time to a significantly lower and relatively stable rate (0.14% per month) at two years. LVEF influenced this risk, and, although the rates of sudden death decreased over time in all patients, patients with a LVEF ≤ 30% were at highest risk of sudden death during this early period, with an estimated sudden death rate of 2.3% in the first month.^21^

Studies with longer-term follow-up have demonstrated a second peak in sudden death incidence, often occurring years after initial MI.^22^ This late increase in sudden death events may be related to progressive ventricular remodeling, recurrent MIs, or new structural heart disease that may alter the cardiac electrophysiology substrate to allow for the onset of sustained ventricular arrhythmias.

## Coronary ischemia and revascularization

The timing of coronary revascularization also modifies the risk of sudden death after MI, and patients with MI who experienced sudden death are less likely to have been revascularized.^23^ The presence of recurrent ischemia itself may therefore be a significant contributor to sudden death in the post-MI period, although in a large analysis of patients with and without left ventricular dysfunction in the Coronary Artery Surgery Study (CASS), the severity of coronary artery disease was not associated with a difference in rates of sudden death versus non-sudden death.^24^ In patients who are revascularized with coronary artery bypass grafting, internal mammary arterial grafts have improved long-term patency and long-term survival rates compared with venous grafts.^25^

Analyses of ICD trials have suggested, however, that ischemia and revascularization may be associated with sudden death and ventricular arrhythmias. In a substudy of the Multicenter Automatic Defibrillator Implantation Trial-II (MADIT-II), no benefit of ICD implantation was seen in patients who were revascularized less than six months prior to enrollment, while there was a 76% reduction in the hazard of sudden death among patients with their most recent revascularization occurring at more than six months before enrollment. Among patients in MADIT-II who did not receive an ICD, the occurrence of sudden death was six times as frequent among patients who underwent coronary revascularization more than six months prior to study enrollment as compared with those who underwent revascularization within six months of enrollment.^26^ Positive stress tests also have been associated with increased ventricular arrhythmias in patients with ICDs.^27^

## Sudden death risk stratification tests

A multitude of tests have been proposed to identify patients at higher risk for the development of sudden death after MI. The assessment of left ventricular function via LVEF has been the most widely used and accepted method of post-MI risk stratification, although, as will be discussed below, when used by itself, LVEF has many limitations as a sudden death risk stratification tool. Other electrocardiographically based non-invasive tests such as microvolt T-wave alternans (TWA), heart rate variability (HRV), signal-averaged electrocardiography (SAECG), and other novel parameters quantify abnormalities in cardiac conduction, repolarization, and/or autonomic tone, which participate in the pathogenesis of sudden death. Invasive studies such as the electrophysiologic study (EPS) can help to identify the presence of a cardiac electrophysiologic substrate favorable for ventricular arrhythmias through ventricular stimulation. Novel myocardial imaging and biochemical assays have also been proposed as markers of increased sudden death risk. These tests and their utility in post-MI sudden death risk stratification are described in detail in the following sections.

### Left ventricular function and morphology

Left ventricular systolic dysfunction (as measured by LVEF) has consistently been a powerful predictor of survival after MI^28,29^ and mortality benefit from ICD implantation.^30,31^ Current guidelines are heavily weighted towards the use of LVEF when selecting candidates for ICD implantation.^32^ When used alone, however, LVEF lacks both sensitivity and specificity for predicting sudden death. Although cardiovascular mortality increases significantly in patients with LVEF < 40%,^28^ in fact, the risk of both sudden death and total mortality increases as LVEF decreases, and there are no data demonstrating that LVEF specifically identifies patients who will experience sudden death.^33^ Patients with severe left ventricular dysfunction are more likely to die of progressive cardiac pump failure or other cardiovascular causes than sudden death.^33,34^ Due to advances in the acute treatment of MI, there are also fewer patients with MI who have severely reduced LVEF. For example, in the Canadian Assessment of Myocardial Infarction (CAMI) study, which was performed in the early 1990s, approximately one-quarter of patients had LVEF < 40%,^28^ while a more recent analysis of patients with acute MI who were all treated with primary PCI revealed that only 12% had LVEF < 40%.^35^

In addition, although patients with significantly reduced LVEF have a higher individual risk of sudden death after MI, in total, more cases of sudden death occur in patients with relatively preserved LVEF after MI.^36,37^ The VALIANT trial demonstrated that while the risk of sudden death was greatest in patients with more severe left ventricular dysfunction (LVEF ≤ 30%), approximately 50% of all sudden cardiac deaths (SCDs) occurred in patients with LVEF > 30%.^21^ Additionally, because significantly more patients had LVEF > 30%, despite the relative risk of SCD being higher in patients with LVEF ≤ 30%, the total number of patients at risk for SCD events was still higher in the group with relatively preserved LVEF. LVEF may also change over time, especially during the early post-MI period when it may be transiently reduced due to myocardial stunning and, with medical therapy and revascularization, left ventricular function may recover significantly after MI. In fact, Ottervanger et al. demonstrated that among ST-elevation MI (STEMI) patients with LVEF ≤ 40% on the third day after MI, one-quarter demonstrated a LVEF improvement to > 40% six months after MI.^38^

In addition to LVEF, other indices of myocardial morphology and remodeling such as left ventricular sphericity index (the ratio of left ventricular end-diastolic volume to the volume of a sphere with the diameter of the left ventricular end-diastolic dimension)^39^ and other novel measures of left ventricular structure obtained via cardiac magnetic resonance imaging,^40^ may have prognostic significance beyond LVEF in predicting sudden death after MI, although these methods will require prospective validation before they can be incorporated into clinical practice.

### Ventricular ectopy and non-sustained ventricular tachycardia

In the reperfusion era, ventricular ectopy and non-sustained VT (NSVT) occurs in 6% to 50% of patients post-MI,^41–44^ and isolated ventricular ectopy is even more common. Studies have demonstrated an association between frequent ventricular ectopy and NSVT and post-MI mortality,^45^ and historically, NSVT has been an important inclusion criterion in some of the early post-MI ICD trials.^46,47^ The presence of ventricular ectopy and NSVT is associated with LVEF,^43^ and therefore shares similar issues with sensitivity and specificity for sudden death prediction and may simply identify a group of patients with more severe heart failure who are also at increased risk of non-sudden death. In the Metabolic Efficiency with Ranolazine for Less Ischemia in Non-ST Elevation Acute Coronary Syndrome-Thrombolysis in Myocardial Infarction (MERLIN-TIMI 36) trial, NSVT was assessed with continuous electrocardiogram (ECG) recordings in the first week after NSTEMI in over 6,300 patients. Over 56% of patients had some VT lasting at least three beats. Episodes of NSVT lasting four to seven beats were present in 18.5% of patients, and were associated with an adjusted hazard ratio (HR) of 2.3 [95% confidence interval (CI) 1.5–3.7; p < 0.001] for sudden death at one year, while episodes of NSVT lasting ≥ eight beats were present in 6.8% of patients and were associated with an adjusted HR of 2.8 (95% CI: 1.5–5.1; p = 0.001) for sudden death at one year. NSVT lasting three beats and NSVT occurring within the first 48 hours after MI were not associated with sudden death.^44^ In a Finnish study of 700 post-MI patients, NSVT was independently associated with sudden death (HR: 4.1, 95% CI: 1.3–13.0; p < 0.01) but was associated with a very poor positive predictive value of only 12%.^6^

In a subanalysis of the Platelet Inhibition and Patient Outcomes (PLATO) trial, however, continuous ECG monitoring one week after acute MI demonstrated that NSVT was associated with an increased risk of cardiovascular death, but not with sudden death.^48^ Many other studies have also demonstrated an association between NSVT and mortality, but not specifically with sudden death.^49–51^ Overall, the use of NSVT in the risk stratification of sudden death post-MI is therefore limited by low sensitivity and specificity for sudden death; it is present in many patients post-MI and, although it is a marker of increased risk of total mortality, it fails to accurately identify those who will die suddenly.

### Electrophysiologic study

Multiple studies have established an association between inducible sustained monomorphic VT and sudden death,^52–57^ and randomized trials have validated the use of the EPS for risk stratification to guide ICD implantation post-MI.^46,47^ Although inducible monomorphic VT is prognostically important after MI, the induction of polymorphic VT, ventricular flutter, or VF is not associated with increased risk of sudden death.^52^ Unfortunately, EPS protocols and criteria for a ‘‘positive’’ study have not been standardized across studies, and this makes the direct comparison of results more complicated.

The MADIT-I trial demonstrated that patients with prior MI, LVEF ≤ 35%, NSVT, and inducible sustained VT on EPS who were randomized to ICD implantation had a 64% reduction in the hazard of total mortality in comparison with conventional/medical therapy alone.^47^ Furthermore, the Multicenter Unsustained Tachycardia Trial (MUSTT)—which included patients with prior MI, LVEF ≤ 40%, and NSVT—demonstrated that inducible VT was associated with a significant increased risk of arrhythmic death or cardiac arrest (18% versus 12% at two years for patients with and without inducible VT, respectively), and that the risk of sudden/arrhythmic death exceeded the risk of non-sudden death. In patients with inducible VT, ICD implantation was associated with a 76% reduction in the relative risk of cardiac arrest or fatal arrhythmia.^46^ Although patients with a negative EPS still had a significant rate of sudden death in MUSTT, relatively few subjects in the study were on β-blockers or angiotensin-converting-enzyme inhibitors, and in a more recent study that evaluated the utility of the EPS in the post-MI population (mean LVEF: 27% ± 7%) in which evidence-based medications were more commonly used, the negative predictive value of the EPS for subsequent arrhythmic death or ICD shock was approximately 96%.^58^

Similarly, in a substudy of the Cardiac Arrhythmias and Risk Stratification after Acute Myocardial Infarction (CARISMA) trial, in 312 patients with prior MI and LVEF ≤ 40%, failure to induce monomorphic VT with EPS six weeks after MI was associated with a 96% negative predictive value for the endpoint of ECG documented VT or VF.^59^ In a substudy of 593 patients in the MADIT-II study, however, although a positive EPS was associated with increased rates of ICD therapy for VT, approximately one-quarter of patients with a negative EPS experienced VT/VF treated by their ICD.^60^

A patient’s LVEF has important effects on the interpretation of results from an EPS. In a substudy of MUSTT that evaluated patients who had negative EPS, the rate of arrhythmic death or cardiac arrest at two years was 8% in patients with an LVEF of 30% to 40%, but significantly higher at 15% in patients with an LVEF of < 30%.^61^ These observations may be explained by considering that patients with lower LVEF may have more clinical heart failure and, as noted above, heart failure itself may contribute to ventricular arrhythmogenesis via multiple mechanisms.

Recently, there has been renewed interest in using EPS for sudden death risk stratification in the immediate post-MI period. In a small Australian study of patients with STEMI and LVEF ≤ 40% within four days of their MI, EPS was performed, and patients with inducible monomorphic VT subsequently were implanted with ICDs. Outcomes in these patients were compared with those of STEMI patients who had LVEF > 40%. The investigators found no difference in the endpoint of death or ventricular arrhythmia in patients with LVEF > 40% and those with LVEF ≤ 40% and a negative EPS (92% versus 93% at four years, respectively), again reinforcing the utility of a negative EPS in selecting a lower risk group of post-MI patients who may not derive significant benefit from ICD implantation.^62^ The Programmed Ventricular Stimulation to Risk Stratify for Early Cardioverter-Defibrillator Implantation to Prevent Tachyarrhythmias following Acute Myocardial Infarction (PROTECT-ICD) trial is an ongoing multicenter randomized controlled trial assessing the role of EPS to guide primary prevention ICD implantation within the first 40 days after MI.^63^

Widespread use of EPS for risk stratification post-MI is limited because it is invasive, associated with procedural risk, and requires specialized equipment. As noted, a negative EPS also does not mean that the future risk of ventricular arrhythmias is non-existent. The EPS therefore may be most useful to refine sudden death risk in combination with other non-invasive tests (see the sections below). In the Alternans Before Cardioverter-Defibrillator (ABCD) trial, EPS had a relatively poor positive predictive value (11%) but an excellent negative predictive value (95%) for sudden death or ICD therapy at one year. Risk was especially elevated in patients who had both positive TWA and positive EPS in comparison with those with negative TWA and negative EPS (11.1% versus 2.3% at one year; p = 0.017). Patients with discordant TWA and EPS results had intermediate rates of sudden death or ICD therapy at one year (6.5%–7.8%). This discordance between EPS and TWA suggests that these tests may reflect different abnormalities in electrophysiologic substrate.^64^

### Microvolt T-wave alternans

TWA refers to beat-to-beat variability in the timing or morphology of T-waves on the surface ECG due to abnormalities in intracellular calcium signaling, which result in heterogeneity in action potential duration and morphology.^65,66^ Increased TWA has been associated with an increased risk for ventricular arrhythmias and has therefore been investigated as a marker of increased sudden death risk.^67^

The ABCD trial demonstrated that in post-MI patients with LVEF ≤ 40% (mean LVEF: 28%), abnormal TWA and invasive EPS had similar positive predictive values (9% versus 11% for TWA and EPS, respectively) and negative predictive values (95% for both) for sudden death or appropriate ICD therapies and, as noted above, the combination of TWA and EPS further refined patient risk.^68^ However, in the Microvolt T-wave Alternans Testing for Risk Stratification of Post-Myocardial Infarction Patients (MASTER) trial, which enrolled 575 patients with prior MI and LVEF ≤ 30% with an ICD, TWA was positive in 51% of patients, and was not associated with the outcome of sudden death or appropriate ICD therapies, although there was an association between abnormal TWA and increased total mortality.^69^ A substudy of the Sudden Cardiac Death in Heart Failure Trial (SCD-HeFT) included a mixed population of infarct-related cardiomyopathy (52% of study population) and non-ischemic cardiomyopathy with LVEF ≤ 35%, but similarly demonstrated no association between TWA and sudden death, appropriate ICD shocks, or total mortality.^70^

TWA, however, may have a role in the risk stratification of patients with relatively preserved LVEF after MI. The Risk Estimation Following Infarction Non-Invasive Evaluation (REFINE) study evaluated TWA two to four weeks and 10 to 14 weeks after MI in 322 patients with relatively preserved LVEF (LVEF < 50% was an inclusion criterion; median LVEF was 40% within one week of the index MI and 47% eight weeks after the index MI). TWA assessed two to four weeks after MI had no association with subsequent cardiac death or cardiac arrest. However, TWA assessed by either exercise or on Holter monitoring 10 to 14 weeks after MI was associated with cardiac death or cardiac arrest [HR: 2.75 (95% CI: 1.08–7.02; p = 0.034) for exercise TWA and HR of 2.94 (95% CI: 1.10–7.87; p = 0.031) for Holter TWA]. Abnormal TWA (both exercise and Holter) at 10 to 14 weeks was associated with a relatively poor sensitivity (45%) and positive predictive value (23%), but with improved specificity (86%) and a high negative predictive value (96%) for cardiac death or cardiac arrest, although the area under the receiver operating curve was relatively poor at 0.65.^71^ Similarly, in a study involving 1,041 patients with LVEF ≥ 40% after MI, TWA was assessed 48 ± 66 days after MI and was associated with an adjusted HR of 19.7 (95% CI: 5.5–70.4; p < 0.0001) and a negative predictive value of 99.6% for the outcome of sudden death or life-threatening arrhythmias.^72^ Therefore, although TWA appears to have limited use in the risk stratification of post-MI patients with LVEF < 30%, there may be a role for TWA testing in patients who have relatively preserved LVEF.

### Measures of autonomic tone: heart rate variability, heart rate turbulence, and baroreflex sensitivity

The autonomic nervous system is critically involved in the pathogenesis of ventricular arrhythmias, and there is an association between increased sympathetic tone and/or reduced parasympathetic activity with greater risk for VT/VF.^73^ Multiple parameters that reflect the sympathetic/parasympathetic balance have therefore been proposed as sudden death risk stratification tools. Heart rate variability (HRV) measures beat-to-beat variation in RR intervals from long-term ECG recordings,^74,75^ while heart rate turbulence (HRT) refers to the presence or absence of the normal acceleration in heart rate following a premature ventricular contraction.^76^ Baroreflex sensitivity (BRS) reflects the normal decrease in heart rate after an increase in blood pressure.^77^ Reductions in HRV, HRT, and BRS all reflect excess sympathetic tone.

The Autonomic Tone and Reflexes After Myocardial Infarction (ATRAMI) trial assessed BRS and HRV in 1,071 post-MI patients, 85.4% of whom had LVEF ≥ 35% (mean LVEF: 49% ± 11%). Among all patients, impaired BRS was associated with a relative risk (RR) of 2.1 (95% CI: 1.1–4.2; p = 0.03) and reduced HRV was associated with an RR of 3.2 (95% CI: 1.6–6.3; p < 0.001) for cardiac mortality. The combination of multiple abnormal parameters was associated with an even greater risk. In patients with LVEF < 35%, HRV had no association with total mortality, although abnormal BRS retained its association with total mortality [RR: 2.8 (95% CI: 1.01–7.72; p = 0.04)]. Patients with LVEF < 35% and abnormal HRV also had a significant increase in the risk of arrhythmic events [RR: 4.1 (95% CI: 1.3–12.2; p = 0.013)], and the combination of LVEF < 35% and abnormal BRS had an even stronger association with arrhythmic events [RR: 6.7 (95% CI: 2.9–15.5; p < 0.001)].^49^ However, another study of 700 Finnish patients with MI in whom HRV and BRS were measured one to two weeks after MI found no association between these parameters and SCD or arrhythmic events.^6^ These disparate results can possibly be explained by differences in the use of β-blockers, which also modulate autonomic influences on the cardiovascular system; in ATRAMI, only 20% of patients were on β-blockers,^78^ while 95% of patients in the above Finnish study, which found no association between HRV and BRS and arrhythmic events, were on β-blockers.^6^

The REFINE trial demonstrated that although HRV, HRT, and BRS had no significant association with cardiac death or cardiac arrest when assessed early after MI (2–4 weeks), assessment at 10 to 14 weeks was predictive of cardiac death or cardiac arrest. When HRV was assessed at 10 to 14 weeks after MI, there was a non-significant trend towards increased adverse events (HR: 2.15; p = 0.066). Similar to TWA, a later assessment of HRT [HR: 2.91 (95% CI: 1.13–7.48; p = 0.026)] and BRS [HR: 2.71 (95% CI: 1.10–6.67; p = 0.030)] were associated with a significantly increased risk of the composite outcome of cardiac death and cardiac arrest.

In an analysis of 244 post-MI patients with LVEF > 35% (mean LVEF: 54% ± 8%), abnormal BRS was associated with a striking increase in cardiovascular mortality at five years (26% versus 2.4% in patients with and without abnormal BRS, respectively). Among patients aged < 65 years, abnormal BRS was associated with an almost 20-fold increase in the relative risk of cardiovascular mortality [RR: 19.6 (95% CI: 4.1–94.8; p = 0.0002)]. Notably, LVEF alone was not a predictor of cardiovascular mortality in this population with relatively preserved left ventricular function.^58^

### Signal-averaged electrocardiography

By utilizing signal filtering and signal averaging techniques to remove noise and detect very low-amplitude (microvolt level) signals at the end of the QRS complex, SAECG identifies the presence of slow or delayed ventricular activation (ventricular ‘‘late potentials’’). These late potentials, which cannot be detected on a standard ECG, may indicate the presence of a substrate favorable for reentrant ventricular tachyarrhythmias. Instead of the standard 12-ECG leads, SAECG utilizes orthogonal ECG leads recorded in the x-, y-, and z-axes. Once orthogonal ECG leads are recorded, the vector magnitude 
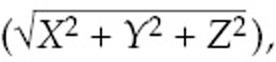
 which is also referred to as the ‘‘filtered QRS complex,’’ is calculated and analyzed. The duration of the filtered QRS complex being > 114 ms, the duration of low amplitude signals in the terminal part of the filtered QRS complex that are ≤ 40 µV being > 38 ms, and the root mean square voltage in the terminal 40 ms of the filtered QRS complex being < 20 µV, all define SAECG abnormalities indicative of the presence of late potentials.^79^

Early studies demonstrated an association between abnormal SAECG and the inducibility of VT at EPS, and that SAECG performed better than LVEF in predicting VT inducibility.^80^ In a subanalysis of MUSTT, an abnormal SAECG was associated with increased rates of cardiac mortality and arrhythmic death or cardiac arrest at five years. The subgroup of patients with abnormal SAECG and LVEF < 30% had a particularly elevated risk of arrhythmic death or cardiac arrest. However, an abnormal SAECG was a stronger predictor of cardiac death than arrhythmic death,^81^ reinforcing its lack of specificity for sudden death.

Revascularization itself may reduce the incidence of late potentials,^82^ and in a recent study of 1,800 patients who were revascularized as treatment for acute MI, SAECG findings were abnormal in 9.3% of patients, but had no association with cardiac death or arrhythmic events.^83^ Multiple studies have evaluated SAECG as a post-MI risk stratification tool, and in a meta-analysis of 9,883 patients, SAECG had a modest sensitivity (62.4%) and specificity (77.4%) for major arrhythmic events (composite of sudden death, resuscitated sudden death, and VT/VF).^84^ When used alone, the SAECG therefore has limited utility in sudden death risk stratification post-MI.

### Standard electrocardiogram parameters

The standard 12-lead ECG is ubiquitous, inexpensive, and does not require long-term monitoring, specialized equipment, or specialized signal processing to extract information that may be useful in sudden death risk stratification. Various markers obtained from a 12-lead ECG have been evaluated as markers of sudden death post-MI. In patients with coronary artery disease, increased QRS duration and bundle branch block (BBB) are associated with more severe coronary artery disease, more severe left ventricular systolic dysfunction, and increased mortality.^85^ Left BBB (but not right BBB) has been associated with total mortality in the CASS Registry^85^ and the MUSTT trial.^86^ Most studies, however, have not demonstrated an association between QRS duration/BBB and sudden death or arrhythmias. Although a substudy of MADIT-II demonstrated that QRS duration > 150 ms was associated with ICD benefit, other studies, including MUSTT, have not found an association between QRS duration/morphology and inducible VT or ventricular arrhythmias.^86,87^ In the VALIANT trial, QRS duration was associated with larger LV volumes and lower LVEF, but was not independently associated with sudden death.^88^ Prolonged QRS and BBB are therefore reflections of more severe ventricular dysfunction instead of markers specifically for increased risk of ventricular arrhythmias or sudden death.

A substudy of MUSTT patients who did not receive antiarrhythmic medications or ICDs demonstrated that ECG left ventricular hypertrophy (LVH) was independently associated with arrhythmic mortality (HR: 1.35; 95% CI: 1.08–1.69). Additionally, ECG-LVH had no association with total mortality, suggesting a mechanistic association with ventricular arrhythmias. Imaging is much more sensitive for the detection of LVH than is the ECG^89^ and, although it warrants further study in post-MI patients, and population-based studies have also found an association between left ventricular mass/hypertrophy and sudden death,^90^ current data do not support the routine use of ECG-LVH for sudden death risk stratification post-MI.

Prolongation of the QT interval, which reflects total time of cardiac depolarization and repolarization, has been associated with sudden death in patients with prior MI.^91^ QT dispersion, which is a measure of the difference between the longest and shortest QT interval measured in all 12 ECG leads, was initially a promising marker of sudden death risk as it was thought to be a surrogate measure of myocardial electrical heterogeneity, which is directly related with ventricular arrhythmias.^92,93^ In a small study of 36 patients with prior MI and NSVT with a mean LVEF of 36%, QT dispersion was significantly higher in patients with inducible VT than it was in patients with negative EPS.^94^ Recent studies, however, have demonstrated that there is a wide overlap of QT dispersion values in normal/healthy patients and in those with prior MI (and other cardiovascular disease states), and in patients with and without ventricular arrhythmias,^95^ and subsequent studies of post-MI patients have demonstrated no significant associations between QT dispersion and mortality and VT/VF.^96^ Furthermore, vectorcardiographic analyses have demonstrated that QT dispersion is primarily related to three-dimensional T-wave loop morphology and measurement error, rather than true dispersion of refractoriness.^95^ As a result, QT dispersion has limited utility in risk stratification post-MI. Other analyses of T-wave morphology, such as the time between the onset of T-wave onset and T-wave peak, have also not demonstrated a significant association with arrhythmic outcomes.^96^

### Novel markers of myocardial electrical heterogeneity

The entire ventricular myocardium does not depolarize or repolarize simultaneously, and some degree of ‘‘electrical heterogeneity’’ is required for normal cardiac function. Heterogeneity in different parts of the right and left ventricles (eg, apical versus basal, right ventricle versus left ventricle, endocardium versus epicardium) results from differences in gene expression during development, varied embryonic origins of different parts of the fully developed heart, and variations in ion channel and gap junction expression resulting in differences in action potential morphology and duration throughout the ventricular myocardium.^92, 97, 98^ Although some degree of electrical heterogeneity is physiological and may have some antiarrhythmic properties, excessive amounts of electrical heterogeneity can be highly proarrhythmic.^93, 99^

ECG markers of myocardial electrical heterogeneity have recently been evaluated for their ability to assess the risk of ventricular arrhythmias and sudden death in various populations. Novel methods of measuring R-wave and T-wave heterogeneity assess the degree to which the R-wave and/or T-wave in a single or in multiple ECG leads deviates from the average R-wave or T-wave, respectively.^100^ Although this methodology has not been directly studied in a large population of post-MI patients, population-based studies have suggested that elevations in R-wave and T-wave heterogeneity are associated with sudden death, and this may prove to be a useful risk stratification tool in post-MI patients in the future.^101^

The spatial ventricular gradient (SVG) is another method available to assess myocardial electrical heterogeneity and its link with ventricular arrhythmias and sudden death. The SVG is a vectorcardiographically derived parameter that is the sum of a vector representing the area of the QRS complex and a vector representing the area of the T-wave (in the x-, y-, and z-axes). A detailed review of the spatial ventricular gradient can be found elsewhere.^102^ The SVG is attractive as a measure of electrical heterogeneity because it has been shown that the SVG reflects the global effect of local variations in repolarization/repolarization across the entire ventricular myocardium, and that the SVG vector points toward the area of the myocardium with the shortest duration of the excited state.^102^ This concept was later extended to the QRS-T angle, the three-dimensional angle between the QRS and T vectors,^102^ and the sum absolute QRST integral (SAI QRST), which is a scalar analog of the SVG calculated as the scalar sum of the areas of the QRS-T complex in the x-, y-, and z-axes.^104^

The SVG vector has not yet been tested prospectively as a risk stratification tool in post-MI patients, although it has been associated with sudden death in the general population.^105^ In a subanalysis of the MADIT-II trial, increased SAI QRST was associated with increased rates of appropriate ICD therapies for VT/VF or sudden death (HR: 1.33 per 100 mV*ms; p = 0.002), although the HR was similar for total-mortality (HR: 1.27 per 100 mV*ms; p = 0.022).^106^ In the prospective PROSE-ICD study, a low SAI QRST was associated with a threefold higher risk of appropriate ICD therapies for VT/VF.^107^ SAI QRST has also been associated with sudden death in the general population.^105^ The disparate results from the MADIT-II and PROSE-ICD studies may reflect differences in the patient populations being studied, as population studies have demonstrated significant interaction with age, gender, and race.^105^ Similarly, QRS-T angle has been associated with appropriate ICD therapies in a post-MI population^108^ and in the general population,^105^ but further research is necessary before SAI QRST and QRS-T angle can be adopted into clinical practice as sudden death risk stratification tools.

### Cardiac biomarkers—brain natriuretic peptide

B-type natriuretic peptide (BNP) and N-terminal-proBNP (NT-proBNP) are secreted by cardiac myocytes in response to hemodynamic stress and other non-cardiovascular stimuli,^109,110^ and are involved in diuresis, natriuresis, and systemic vasodilation.^110^ In a study of post-MI patients with very high rates of β-blocker utilization (97%), BNP measured before hospital discharge was independently associated with sudden death even after adjusting for LVEF and New York Heart Association (NYHA) functional class [patients in the highest quartile of BNP had an adjusted HR of 3.4 (p = 0.037) for sudden death as compared with other patients]. Notably, the adjusted HR for sudden death was similar for LVEF < 30% (adjusted HR: 3.7; p = 0.047). A BNP value in the highest quartile had a specificity of 75.8% and a sensitivity of 53.3% for sudden death, with a low positive predictive value of 6.2% and a high negative predictive value of 98.2%. Importantly, although LVEF < 30% was also associated with a similar risk of non-sudden cardiac death (adjusted HR: 3.5; p = 0.025), BNP had no association with non-sudden cardiac death.^111^ BNP levels have been shown to be strong, independent predictors of sudden death in other study populations, including post-MI and non-ischemic causes of heart failure as well.^112^

In a small study in which NT-proBNP was assessed prior to ICD implantation in patients with MI and LVEF ≤ 30%, a NT-proBNP level > 2,536 pg/ml was independently associated with appropriate ICD therapies for ventricular arrhythmias over one year of follow-up [RR: 7.7; (p = 0.024)], even after adjustment for LVEF, NYHA functional class, and multiple other covariates.^113^ In another retrospective study evaluating the associations between BNP, ventricular arrhythmias, and total mortality in ICD recipients, elevated BNP and NT-proBNP levels were independently associated with appropriate ICD therapies for ventricular arrhythmias, and the risk of arrhythmia significantly exceeded the risk for total mortality.^114^ Multiple other studies have linked elevated BNP levels post-MI to other adverse cardiovascular factors, such as total mortality and clinical heart failure.^115–117^

Whether the association between BNP, arrhythmias, and sudden death is related to adverse hemodynamic factors, ventricular remodeling, and/or other factors that promote arrhythmogenesis is unclear at this point in time. Like many other potential risk stratification tools, natriuretic peptides require prospective evaluation for risk stratification post-MI before they can be adopted into routine clinical practice.

## Timing of risk stratification post-myocardial infarction

The optimal time to perform risk stratification testing after MI is currently unanswered, but studies have suggested that risk stratification in the early period after MI is less useful than risk stratification performed later on. This was demonstrated in the REFINE study, where HRT, BRS, and TWA assessed at two to four weeks after MI had no association with subsequent outcomes, while later, assessments of these parameters at 10 to 14 weeks were associated with cardiac death or cardiac arrest.^71^ This result is not necessarily surprising, as myocardial remodeling after infarction takes time, and acute derangements in the cardiovascular system post-MI, including the degree of left ventricular systolic dysfunction, can change/improve over time. The increasing proportion of non-arrhythmic sudden death in the early post-MI period (due to complications such as free wall rupture) also likely influences this observation. Studies that investigated the utility of primary prevention ICD implantation in patients within the first 40 days after MI have similarly demonstrated no mortality benefit with regards to ICD implantation, even in the setting of other abnormal non-invasive testing.^118,119^ A multicenter, prospective, randomized control trial, PROTECT-ICD, investigating the utility of EPS-guided ICD implantation within the first 40 days after MI is currently underway.^63^ Our understanding of the optimal timing for invasive and non-invasive risk stratification testing will likely continue to evolve over time.

## Sudden death risk stratification models

In general, single tests lack both sensitivity and specificity for predicting sudden death. Assessing multiple tests simultaneously, however, shows more promise, and studies such as ATRAMI, ABCD, and REFINE have demonstrated improved discrimination for an arrhythmic substrate post-MI by performing multiple tests that evaluate different aspects of arrhythmogenesis simultaneously. These studies demonstrated that arrhythmic risk was very low for patients with no risk factors post-MI, and that risk increased as multiple parameters were abnormal. Investigators have extended this concept to create risk stratification models that are useful for predicting arrhythmic mortality and total mortality, and therefore the benefit associated with ICD implantation given that ICDs can only prevent arrhythmic-related sudden death.

**Figure 2:**
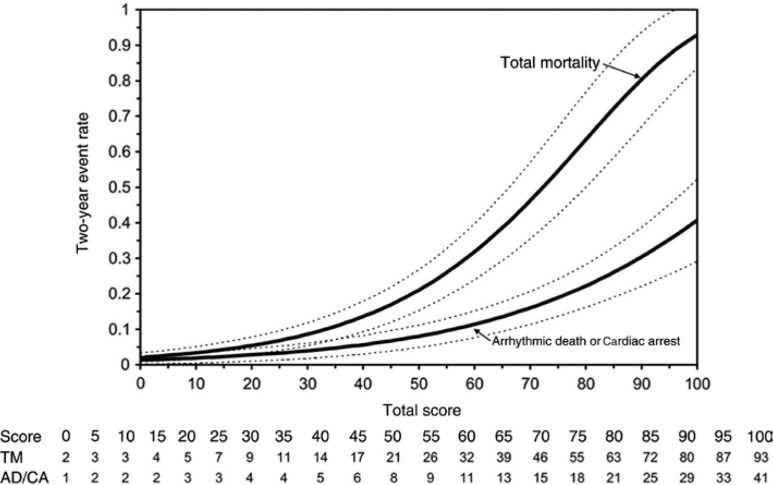
The relationship between risk score and two-year rates of total mortality and arrhythmic death/cardiac arrest in patients in the MUSTT trial. AD/CA: arrhythmic death/cardiac arrest; TM: total mortality. Reproduced with permission from Buxton AE, Lee KL, Hafley GE, et al. Limitations of ejection fraction for prediction of sudden death risk in patients with coronary artery disease. Lessons from the MUSTT study. *J Am Coll Cardiol.* 2007;50:1150–1157.

The MUSTT investigators developed a multivariable model to predict the risk of arrhythmic death/cardiac arrest and total mortality at two years, and found that factors such as inducible VT, clinical heart failure, LVEF, and intraventricular conduction delay/left bundle branch block were associated with both arrhythmic and non-arrhythmic death to different extents **(Table 1)**. The model demonstrated that across all risk strata, the risk of arrhythmic death or cardiac arrest accounted for about half of the total number of deaths. The MUSTT model clearly demonstrates the limitation of using LVEF alone for risk stratification post-MI. For example, a 60-year-old man with an LVEF of 25%, prior coronary artery bypass grafting, and no other risk factors would have a two-year risk of arrhythmic mortality of approximately 2% and a two-year risk of total mortality of 5%, respectively **(Figure 2)**. This predicted two-year mortality is lower than that observed in patients treated with ICDs in MUSTT or MADIT-II.^120^

**Figure 3:**
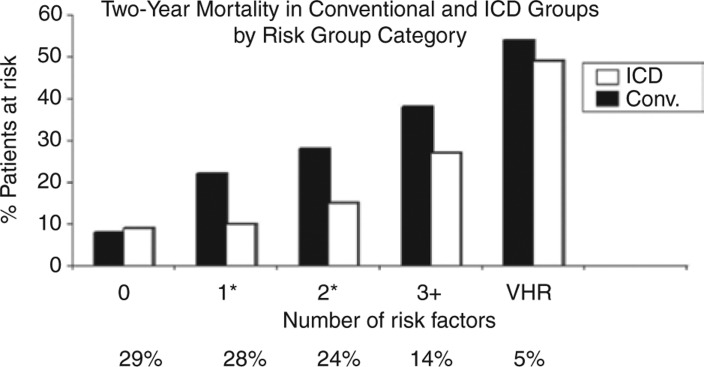
Two-year mortality in patients with and without ICDs in the MADIT-II trial. No significant survival benefit associated with ICD implantation was seen in patients with no risk factors, or in those patients at very high risk due to the presence of severe renal dysfunction. *p < 0.05 for the comparison between conventional therapy (Conv.) and ICD groups. VHR: very high risk. See text for further details. Reproduced with permission from Goldenberg I, Vyas AK, Hall WJ, et al. Risk stratification for primary implantation of a cardioverter-defibrillator in patients with ischemic left ventricular dysfunction. *J Am Coll Cardiol.* 2007;51(3):288–296.

The MADIT-II investigators similarly investigated factors beyond LVEF that contributed to total mortality and ICD benefit. They found that patients with creatinine ≥ 2.5 mg/dl or blood urea nitrogen (BUN) ≥ 50 mg/dl had approximately 50% total mortality at two years, and that they did not derive benefit from ICD implantation. After excluding these ‘‘very-high-risk’’ patients, they then identified five clinical factors associated with mortality: age > 70 years, NYHA class > II, BUN > 26 mg/dl, QRS duration > 120 ms, and the presence of atrial fibrillation. Benefit from ICD implantation was seen in patients with one to two risk factors. However, no significant benefit was seen among patients with no risk factors or with ≥ three risk factors **(Figure 3)**. In ‘‘very-high-risk’’ patients, the rates of non-sudden death were significantly higher than the rates of sudden death, regardless of whether patients were randomized to ICD implantation.^121^

Bilchick et al. evaluated and validated risk factors associated with mortality over one to four years in more than 45,000 patients sourced from multiple ICD registries and, similar to the results from MUSTT and MADIT-II, found that renal dysfunction, LVEF ≤ 20%, age ≥ 75 years, NYHA class > II, and the presence of atrial fibrillation were associated with mortality after ICD implantation. This study also identified diabetes and chronic pulmonary disease as risk factors for mortality after ICD implantation.^122^

The Seattle Heart Failure Model (SHFM; includes the variables of age, gender, systolic blood pressure, ischemic cardiomyopathy, NYHA class, LVEF, use of heart failure medication, and serum sodium and serum creatinine values) was used to assess mortality and ICD benefit in 2,483 SCD-HeFT participants (a mix of post-MI patients and patients with non-ischemic cardiomyopathy, symptomatic heart failure, and LVEF ≤ 35%). Similar to the results from MADIT-II, use of an ICD was not associated with mortality benefit in patients in the highest quintile of risk assigned by the SHFM. The absolute mortality benefit associated with ICD implantation in the remaining quintiles of risk ranged from 6.6% in the first quintile to 14.0% in the fourth quintile.^123^

The capacity of the SHFM was extended with the development of the Seattle Proportional Risk Model. This model was used to evaluate 9,885 patients from multiple prospective heart failure studies, and specifically assessed the relative risks of sudden and non-sudden death according to SHFM risk factors. The analysis revealed that male gender, younger age, lower NYHA class, higher body mass index, absence of diabetes, absence of renal dysfunction, and absence of hyponatremia were associated with a risk of sudden death that was elevated out of proportion to the risk of non-sudden death, while factors such as LVEF were not associated with an elevated risk of sudden versus non-sudden death.^124^

Unfortunately, although these models consistently have similar factors associated with mortality and appear to allow clinicians to more optimally counsel patients on the risk of mortality with and without ICD implantation, they have not yet been prospectively validated in a study of patients with/without ICDs, and therefore have not been incorporated into clinical guidelines.^32^

## Conclusions and the future of sudden death risk stratification after myocardial infarction

Despite improved access to early revascularization and contemporary optimal medical therapy after MI (with novel antiplatelet agents, β-blockers, angiotensin-converting enzyme inhibitors/angiotensin receptor blockers, and aldosterone antagonists, which are clearly associated with reduced mortality after MI), sudden death continues to be the most common mode of death after MI. As demonstrated above, the current paradigm of relying primarily on LVEF to identify ‘‘high-risk’’ patients ideal for ICD implantation is fraught with limitations. Based on the presence or absence of additional risk factors or according to results found via other forms of cardiovascular testing, some patients with very low LVEF may actually have a rather low risk of sudden death, even lower than that of some patients with relatively preserved LVEF and multiple other risk factors. Additionally, some patients with low LVEF after MI may have multiple other comorbidities that significantly attenuate the benefits associated with ICD implantation. Many patients who receive ICDs for the primary prevention of sudden death after an MI also never use their ICD and, given the cost and risk associated with ICD implantation, it is imperative that future studies prospectively validate risk stratification models so that ICDs can be more optimally used. As patients with relatively preserved LVEF (> 40%) after MI still have a significant risk of sudden death, and represent the majority of post-MI patients, studies prospectively evaluating risk stratification in this population are also critically needed.

Aside from LVEF, numerous other SCD risk stratification studies, as mentioned above, have been proposed, yet none have found their way into guidelines or been widely adopted by clinicians. This is due to the fact that these tests are often difficult to obtain, interpret, and understand, because the optimal time to test remains unclear and because prospective studies have not consistently demonstrated excellent test performance. Additionally, studies have evaluated these alternative SCD risk stratification tests at different points in time, and as the use of goal-directed medical therapy and primary PCI have increased, the results of some older studies may not apply in the current era. However, tests other than LVEF may still be useful, especially in those with relatively preserved LVEF after MI, and further prospective study is therefore warranted. Ventricular arrhythmias can arise through multiple mechanisms, and therefore the combination of LVEF and other novel SCD risk stratification tests likely does refine SCD risk, but further prospective evaluation of a strategy of combining LVEF and other SCD risk stratification tests is necessary before these tests can be adopted into routine clinical practice. As long-term cardiac monitoring with implantable loop recorders becomes more ubiquitous, this may also allow for other novel methods of dynamically assessing SCD risk in survivors of MI over very long periods of time to be used.

Above all, clinicians need to be aware that SCD risk exists along a continuum, even among patients with severely reduced LVEF after MI. Risk scores from MADIT-II, MUSTT, and the SHFM can help clinicians look beyond LVEF, and can help provide a more patient-specific risk of SCD and potential benefit of ICD implantation. In select patients, performing additional tests such as an EPS or TWA may also help refine SCD risk. Unfortunately, there will always be uncertainty around an individual patient’s risk of SCD as well as the benefit associated with ICD implantation and, until better prospective risk stratification data become available, clinicians should educate patients about this uncertainty while following currently accepted guidelines for SCD risk stratification and ICD implantation.^32^

## Figures and Tables

**Table 1:** MUSTT Risk Stratification Variables for Total Mortality and Arrhythmic Death.

Characteristic	Points
**Total Mortality Risk Score**	
LVEF ≤ 20%	20
LVEF 20% to 40%	1 point for each LVEF percentage < 40%
LVEF = 40%	0
IVCD/LBBB	12
NYHA functional class II	7
NYHA functional class III	14
Inducible monomorphic VT at EPS	8
Age ≥ 80 years	15
Age 50 to 80 years	0.5 points for each year of age > 50 years
Age ≤ 50 years	0
No prior CABG	7
History of atrial fibrillation	11
History of congestive heart failure	13
**Arrhythmic Death/Cardiac Arrest Score**	
Inducible VT at EPS	17
History of congestive heart failure	19
Patient enrolled as an inpatient	17
LVEF ≤ 20%	20
LVEF 20% to 40%	1 point for each LVEF percentage < 40%
LVEF = 40%	0
NSVT not within 10 days of CABG	17
IVCD/LBBB	10

MUSTT: Multicenter Unsustained Tachycardia Trial; LVEF: left ventricular ejection fraction; IVCD: intraventricular conduction delay; LBBB: left bundle branch block; NYHA: New York Heart Association; VT: ventricular tachycardia; EPS: electrophysiologic study; CABG: coronary artery bypass grafting; NSVT: non-sustained ventricular tachycardia.
